# Tamoxifen retinopathy: a case report

**DOI:** 10.1186/s40064-015-1258-2

**Published:** 2015-09-17

**Authors:** Lingling Wang, Heng Miao, Xiaoxin Li

**Affiliations:** Department of Ophthalmology, Peking University People’s Hospital, Xizhimen South Street 11, 100044 Beijing, China; Key Laboratory of Vision Loss and Restoration, Ministry of Education, Beijing, China; Beijing Key Laboratory of Diagnosis and Therapy of Retinal and Choroid Diseases, Beijing, China

**Keywords:** Tamoxifen, Retinopathy, Spectral-domain optical coherence tomography, Electro-oculogram

## Abstract

**Purpose:**

To present a case of tamoxifen retinopathy on a 57-year-old woman.

**Design:**

An observational case.

**Methods:**

A review of history, clinical examination, and findings on Spectral-domain Optical Coherence Tomography (SD-OCT), fluorescein angiogram (FA) and electro-oculogram (EOG) was conducted.

**Results:**

A 57-year old female patient, who had been treated with oral tamoxifen after mastectomy due to breast cancer, had profound visual impairment in both eyes. Disruption of the ellipsoid zone and interdigitation zone which represent photoreceptor damage as well as macular thinning was revealed on SD-OCT in both eyes. Bilateral retinal pigmented epithelium (RPE) function compromised was indicated by reduced Arden ratio in EOG.

**Conclusion:**

Tamoxifen-induced retinopathy could be detected by SD-OCT and EOG. As it is irreversible, patients who are given tamoxifen need regular ophthalmic consultation, including SD-OCT and EOG before and during the treatment in order to early detect and avoid further retinal damage.

## Background

Tamoxifen is an oral estrogen antagonist drug, which is an adjuvant treatment of breast cancer when used in low doses (Early Breast Cancer Trialists’ Collaborative Group 2005; US Food and Drug Administration, Center for Drug Evaluation and Research 2006). Toxic effects of tamoxifen on ocular were first described in 1978 and the reported incidence of ocular side effects of tamoxifen ranges from 0.9 to 11 %, including keratopathy, cataract, optic neuritis, crystalline retinopathy with or without macular edema, and pseudocysticfoveal cavitation (Kaiser-Kupfer and Lippman 1978; Salomao et al. 2007; Doshi et al. 2014).

The current standard treatment with Tamoxifen for breast cancer is 5 consecutive years, however the global Adjuvant Tamoxifen: Longer against Shorter (ATLAS) trial has recently showed that 10 years of treatment reduced the risk of recurrence and mortality of breast cancer (Davies et al. 2013). Thus, a longer treatment of tamoxifen may be adopted and its toxicity on eyes should be in more concerned. We present a case of tamoxifen retinopathy detected mainly by spectral-domain optical coherence tomography (SD-OCT) and electro-oculogram (EOG).

## Case report

A 57-year-old female presented to our clinic complaining of gradual progressive diminution of vision for 9 years and distortion for 3 months in the left eye. Her best visual acuity had been 20/20 bilaterally in the past. Her past medical history included hypertension, diabetes, hyperlipidemia and a radical mastectomy of the right breast for cancer 13 years ago and subsequent initiation of oral tamoxifen 20 mg per day for 5 year with cumulative dose of 36.5 g. Chemotherapy with paclitaxel and epirubin for breast cancer had been used for 6 months after mastectomy. Current medications were levamlodipinebesylate, olmesartan, acarbose, Novolin 30R, probucol and simvastatin.

**Fig. 1:**
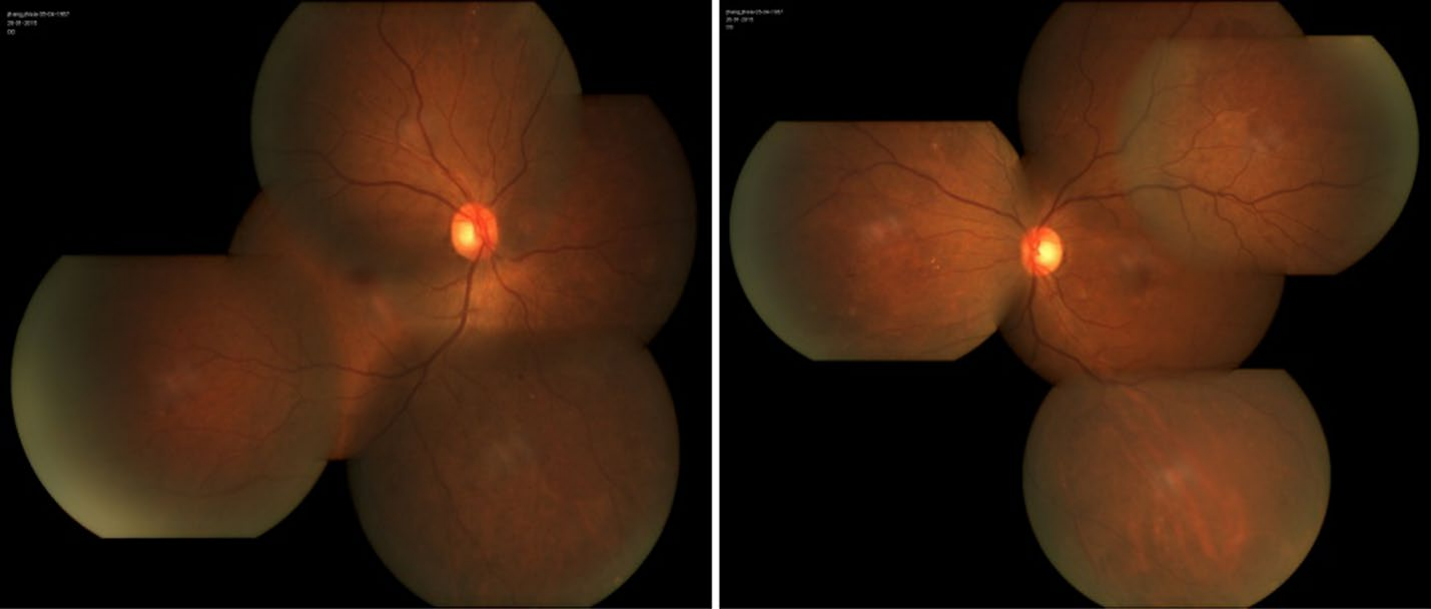
Fundus photographs, *right* and *left*, respectively, revealing the foveal light reflex was dismissed with hard exudates and white refractive deposits in peripheral retina

**Fig. 2:**
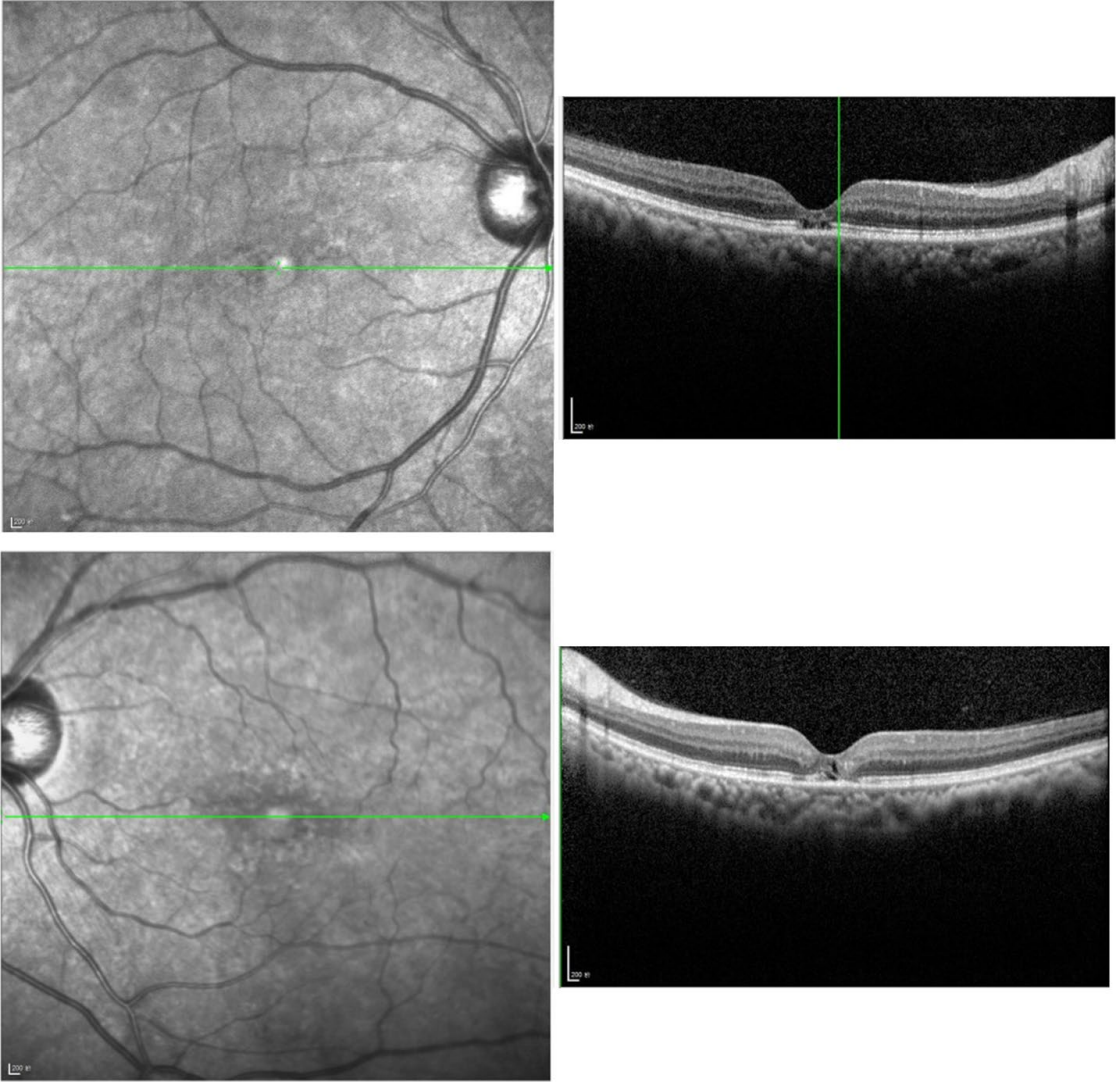
Spectral-domain Optical Coherence Tomography (SD-OCT), showed bilateral disruption of the ellipsoid zone and interdigitation zone

**Fig. 3:**
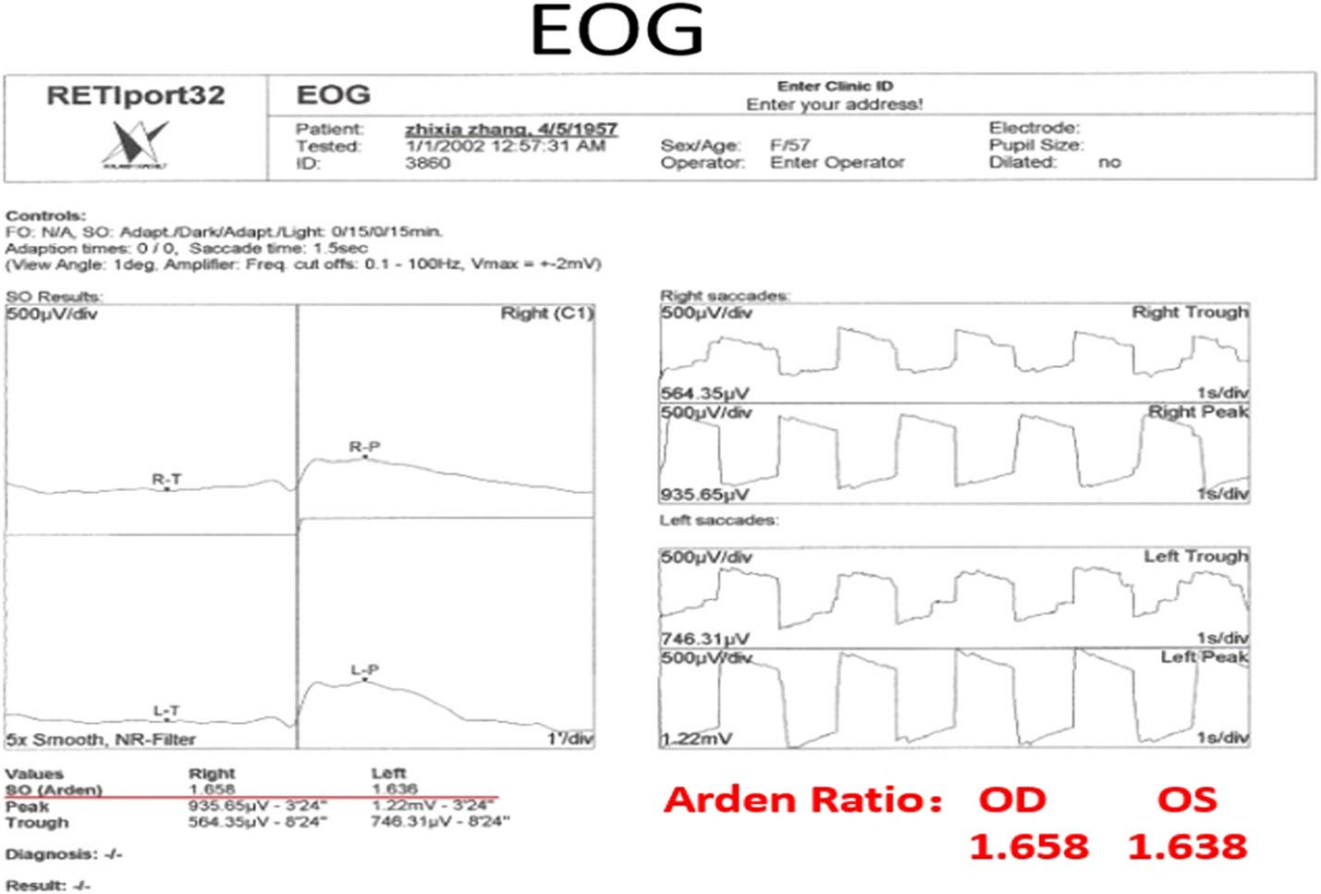
ROG showed that the arden radio was compromised by 1.658 and 1.638 of the right and left eye, respectively

On examination, her best-corrected visualacuity was 20/50 in the right eye and 20/80 in the left eye. External examination and pupillary evaluation were normal. Slit-lam examination revealed clear corneas, quiet anterior chambers and mild nuclear and cortical sclerotic cataracts in both eyes. On dilated fundus examination, the foveal light reflex was dismissed with hard exudates and white refractive deposits in peripheral retina bilaterally (Fig. 1). SD-OCT was performed using OCT SPECTRALIS (Heidelberg Engineering GmbH 69121 Heidelberg/Germany). SD-OCT revealed bilateral disruption of the ellipsoid zone and interdigitation zone which was more diffuse in the left eye (Fig. 2). Bilateral macular thickness was measured with calipers at 119 μm in the right and 156 μm in the left, indicating macular thinning. Fundus fluorescein angiogram showed telangiectasia in the macular zone and scattered microaneurysm. On EOG, Arden radio was compromised by 1.658 and 1.638 of the right and left eye, respectively (Fig. 3). Electro-retinogram, visually evoked potentials and visual fields (Octopus) examination did not disclose obvious abnormities in both eyes.

The patient had once received chemotherapy with paclitaxel and epirubin for 6 months and side effects of these drugs on retina should be considered. It had been reported that paclitaxel could cause macular edema and transient blurred vision with photopia while epirubicin mainly affected the stability of tear film (Li et al. 2014; Seidman et al. 1994; Karamitsos et al. 2013). No retinopathy represented in this case had been reported in patients on the medication of paclitaxel or epirubicin.

None of the patient’s current medication had been reported to be toxic towards retina. So it is highly probable that the patient’s retinopathy was blamed to tamoxifen. Hence, tamoxifen retinopathy and moderate non-proliferated diabetic retinopathy was diagnosed. Oral vitamin C 1 g QD was prescribed.

In follow-up after 3 months by telephone, the patient denied any visual changes.

## Discussion

Tamoxifen retinopathy is characterized by bilateral presence of crystalline deposits with or without macular edema (Drenser et al. 2006). Recently, with developments of retinal imaging such as SD-OCT, more and more manifestations of tamoxifen retinopathy has been reported including cavitation in the macular (Doshi et al. 2014).

In this case, SD-OCT revealed diffused disruption of the ellipsoid zone and interdigitation zone in the left eye, which was correspond to the complaint of blurred vision with distortion in the left eye while no obvious distortion was reported in the right eye. The atrophy of the retinal tissue disclosed by SD-OCT was similar to the previous case reports of tamoxifen-induce retinopathy which presented as cavitation in the macular (Doshi et al. 2014).

EOG is a sensitive method to detect the function of retinal pigmented epithelium (RPE), which plays a critical role in the homeostasis of photoreceptors, phagocytizing the outer segment tips of photoreceptors. It had been found to reveal differences in patients with tamoxifen retinopathy, but it was not statistically significant, possibly owing to limited number of cases (Kuchenbecker et al. 2001). However, our patient’s EOG showed reduced Arden radio, indicating the impaired function of the RPE and explaining why the patient’s disease was still progressing even tamoxifen had been stopped for 8 years. In vitro study had revealed that tamoxifen has potential toxicity on RPE and photoreceptors, as well as alternating Muller cell’s function (Cho et al. 2012; Kim et al. 2014).

A longer treatment of tamoxifen therapy may be adopted according to ATLAS trial (Davies et al. 2013). Meanwhile more attention should be paid on whether it would increase the risk of developing tamoxifen ocular toxicity. Unlike corneal toxicity that has been reported to be reversible if tamoxifen therapy is stopped (Zinchuk et al. 2006), retinopathy is irreversible (Doshi et al. 2014; Nair et al. 2012). So it is of great value to detect signs of tamoxifen-induced retinopathy. As SD-OCT and EOG are sensitive to detect, from anatomical and functional aspects respectively, we suggest patients treated with tamoxifen taking a baseline ophthalmic examination before therapy initiation and periodic evaluations including SD-OCT and EOG during the treatment.
